# 1-(2-Amino-6-methyl­pyrimidin-4-yl)-*N*,*N*-dimethyl­piperidin-4-aminium chloride

**DOI:** 10.1107/S1600536812046533

**Published:** 2012-11-17

**Authors:** S. Sreenivasa, K.E. ManojKumar, P.A. Suchetan, T. Srinivasan, B. S. Palakshamurthy, D. Velmurgan

**Affiliations:** a Department of Studies and Research in Chemistry, Tumkur University, Tumkur, Karnataka 572 103, India; b Department of Studies and Research in Chemistry, U.C.S., Tumkur University, Tumkur, Karnataka 572 103, India; c Centre of Advanced Study in Crystallography and Biophysics, University of Madras Guindy Campus, Chennai 600 025, India; d Department of Studies and Research in Physics, U.C.S., Tumkur University, Tumkur, Karnataka 572 103, India

## Abstract

In the title mol­ecular salt, C_12_H_22_N_5_^+^·Cl^−^, the cation is protonated at the dimethyl-substituted tertiary N atom. The piperidine ring adopts a chair conformation with the exocyclic N—C bond in an equatorial orientation. The dihedral angle between the piperidine ring (all atoms) and the pyrimidine ring is 14.00 (1)°. In the crystal, the ions are connected by N—H⋯N hydrogen bonds, forming inversion dimers, which are further connected by N—H⋯Cl hydrogen bonds. Aromatic π–π stacking inter­actions [centroid–centroid separation = 3.4790 (9) Å] are also observed in the structure.

## Related literature

For background to pyrimidine derivatives and their biological activity, see: Patel *et al.* (2003 ▶).
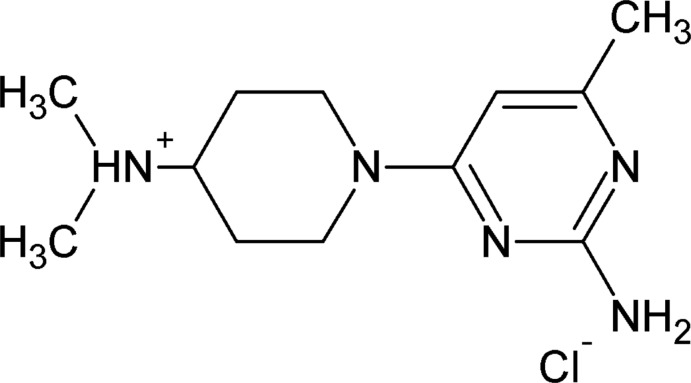


## Experimental

### 

#### Crystal data

C_12_H_22_N_5_^+^·Cl^−^
*M* *_r_* = 271.80
Monoclinic, 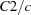

*a* = 24.7908 (12) Å
*b* = 8.2419 (4) Å
*c* = 13.8764 (6) Å
β = 91.968 (2)°
*V* = 2833.6 (2) Å^3^
*Z* = 8
Mo *K*α radiation
μ = 0.26 mm^−1^
*T* = 298 K
0.21 × 0.18 × 0.03 mm

#### Data collection

Bruker APEXII CCD diffractometer
Absorption correction: multi-scan (*SADABS*; Sheldrick, 2004) ▶ *T*_min_ = 0.947, *T*_max_ = 0.994
10807 measured reflections
2502 independent reflections
2266 reflections with *I* > 2σ(*I*)
*R*_int_ = 0.024

#### Refinement

*R*[*F* ^2^ > 2σ(*F* ^2^)] = 0.036
*wR*(*F* ^2^) = 0.107
*S* = 1.08
2502 reflections
176 parameters
H atoms treated by a mixture of independent and constrained refinement
Δρ_max_ = 0.28 e Å^−3^
Δρ_min_ = −0.24 e Å^−3^

### 

Data collection: *APEX2* (Bruker, 2004 ▶); cell refinement: *SAINT-Plus* (Bruker, 2004 ▶); data reduction: *SAINT-Plus*; program(s) used to solve structure: *SHELXS97* (Sheldrick, 2008 ▶); program(s) used to refine structure: *SHELXL97* (Sheldrick, 2008 ▶); molecular graphics: *ORTEP-3* (Farrugia, 2012 ▶); software used to prepare material for publication: *SHELXL97*.

## Supplementary Material

Crystal structure: contains datablock(s) I, global. DOI: 10.1107/S1600536812046533/hb6988sup1.cif

Structure factors: contains datablock(s) I. DOI: 10.1107/S1600536812046533/hb6988Isup2.hkl

Supplementary material file. DOI: 10.1107/S1600536812046533/hb6988Isup3.cml

Additional supplementary materials:  crystallographic information; 3D view; checkCIF report

## Figures and Tables

**Table 1:** Hydrogen-bond geometry (Å, °)

*D*—H⋯*A*	*D*—H	H⋯*A*	*D*⋯*A*	*D*—H⋯*A*
N3—H3*NB*⋯Cl1	0.84 (2)	2.60 (2)	3.4284 (17)	167.2 (17)
N5—H5*N*⋯Cl1^i^	0.878 (19)	2.20 (2)	3.0785 (14)	177.1 (17)
N3—H3*NA*⋯N2	0.86 (2)	2.26 (2)	3.114 (2)	175.5 (18)

Symmetry code: (i) 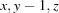.

**Fig. 1.**
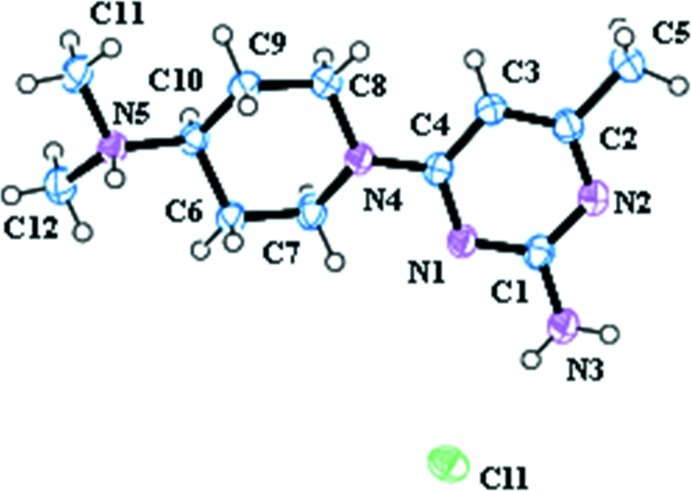
Molecular structure of the title compound, showing displacement ellipsoids drawn at the 50% probability level.

**Fig. 2.**
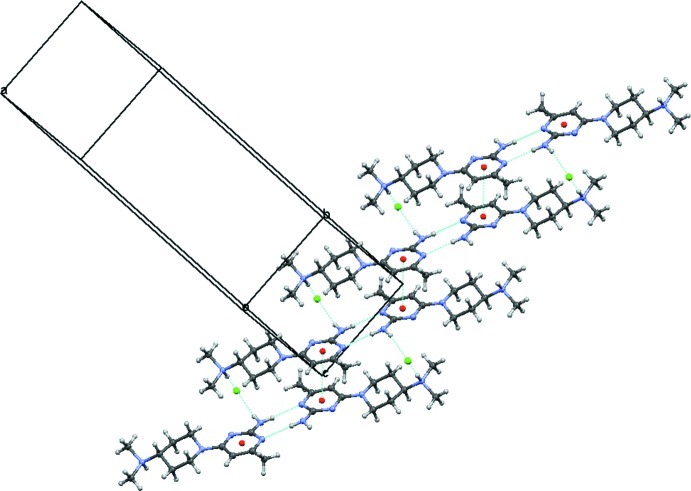
Crystal packing of the title compound. Hydrogen bonds and π-π interactions are shown as dashed lines.

## supplementary crystallographic information

### Comment

Nitrogen-containing heterocyclic ring such as pyrimidine is a promising structural moiety for drug design. Pyrimidine derivatives form a component in a number of useful drugs and are associated with many biological and therapeutical activities (Patel *et al.*, 2003). In this view, we synthesized the title compound to study its crystal structure. The title compound crystallizes in monoclinic *C*2/*c* space group with the piperidine ring in the molecule adopting chair conformation. The dihedral angle between the piperidine ring and the pyrimidine ring in the molecule is 14.00 (1)°. In the crystal structure, the molecules are linked to one another through N—H···N hydrogen bonds generating *R*_2_^2^(8) ring patterns forming inversion related dimers. These dimers are further connected to one another through N—H···Cl hydrogen bonds and weak π-π interactions.

### Experimental

To a solution of 2-amino-4-chloro-6-methylpyrimidine (1.39 mmol) in acetonitrile (3 ml) was added 4-(dimethylamino)piperidine (1.66 mmol), xantphos (0.0695 mmol), Pd(OAc)_2_ (0.139 mmol) and Cs_2_CO_3_ (2.78 mmol). The reaction mixture was irradiated with microwave radiation at 60° C for 1.5 hrs. The reaction was monitored by TLC. The solvent was removed under reduced pressure and the crude product was purified by column chromatography using MDC/methanol as eluent. Colourless prisms were obtained from slow evaporation of the solution of the compound in dilute alcohol.

### Refinement

The H atoms were positioned with idealized geometry using a riding model with C—H = 0.93- 0.97 Å. All C—H atoms were refined with isotropic displacement parameters (set to 1.2–1.5 times of the *U*_eq_ of the parent atom) and N—H atoms were refined freely

### Crystal data

**Table:**

C_12_H_22_N_5_^+^·Cl^−^	*F*(000) = 1168
*M**_r_* = 271.80	Prism
Monoclinic, *C*2/*c*	*D*_x_ = 1.274 Mg m^−^^3^
Hall symbol: -C 2yc	Mo *K*α radiation, λ = 0.71073 Å
*a* = 24.7908 (12) Å	Cell parameters from 2266 reflections
*b* = 8.2419 (4) Å	θ = 1.6–52°
*c* = 13.8764 (6) Å	µ = 0.26 mm^−^^1^
β = 91.968 (2)°	*T* = 298 K
*V* = 2833.6 (2) Å^3^	Prism, colourless
*Z* = 8	0.21 × 0.18 × 0.03 mm

### Data collection

**Table:**

Bruker APEXII CCD diffractometer	2502 independent reflections
Radiation source: fine-focus sealed tube	2266 reflections with *I* > 2σ(*I*)
Graphite monochromator	*R*_int_ = 0.024
Detector resolution: 1.20 pixels mm^-1^	θ_max_ = 25.0°, θ_min_ = 1.6°
ω scans	*h* = −28→29
Absorption correction: multi-scan (*SADABS*; Sheldrick, 2004)	*k* = −9→8
*T*_min_ = 0.947, *T*_max_ = 0.994	*l* = −16→16
10807 measured reflections	

### Refinement

**Table:**

Refinement on *F*^2^	Primary atom site location: structure-invariant direct methods
Least-squares matrix: full	Secondary atom site location: difference Fourier map
*R*[*F*^2^ > 2σ(*F*^2^)] = 0.036	Hydrogen site location: inferred from neighbouring sites
*wR*(*F*^2^) = 0.107	H atoms treated by a mixture of independent and constrained refinement
*S* = 1.08	*w* = 1/[σ^2^(*F*_o_^2^) + (0.0623*P*)^2^ + 1.9022*P*] where *P* = (*F*_o_^2^ + 2*F*_c_^2^)/3
2502 reflections	(Δ/σ)_max_ = 0.032
176 parameters	Δρ_max_ = 0.28 e Å^−^^3^
0 restraints	Δρ_min_ = −0.24 e Å^−^^3^
0 constraints	

### Special details

**Table:**

Geometry. All e.s.d.'s (except the e.s.d. in the dihedral angle between two l.s. planes) are estimated using the full covariance matrix. The cell e.s.d.'s are taken into account individually in the estimation of e.s.d.'s in distances, angles and torsion angles; correlations between e.s.d.'s in cell parameters are only used when they are defined by crystal symmetry. An approximate (isotropic) treatment of cell e.s.d.'s is used for estimating e.s.d.'s involving l.s. planes.
Refinement. Refinement of *F*^2^ against ALL reflections. The weighted *R*-factor *wR* and goodness of fit *S* are based on *F*^2^, conventional *R*-factors *R* are based on *F*, with *F* set to zero for negative *F*^2^. The threshold expression of *F*^2^ > σ(*F*^2^) is used only for calculating *R*-factors(gt) *etc*. and is not relevant to the choice of reflections for refinement. *R*-factors based on *F*^2^ are statistically about twice as large as those based on *F*, and *R*- factors based on ALL data will be even larger.

### Fractional atomic coordinates and isotropic or equivalent isotropic displacement parameters (Å^2^)

**Table:**

	*x*	*y*	*z*	*U*_iso_*/*U*_eq_	
Cl1	0.199032 (17)	1.43859 (6)	1.09649 (3)	0.04241 (17)	
H5N	0.2006 (7)	0.482 (2)	0.9400 (14)	0.034 (5)*	
H3NA	0.0476 (8)	1.470 (3)	1.0540 (14)	0.044 (5)*	
H3NB	0.0967 (9)	1.378 (2)	1.0672 (14)	0.038 (5)*	
N1	0.07954 (5)	1.14699 (15)	0.96467 (10)	0.0313 (3)	
N5	0.20241 (5)	0.49472 (15)	0.87744 (10)	0.0286 (3)	
N2	−0.00534 (5)	1.28695 (16)	0.95048 (10)	0.0350 (3)	
N4	0.09426 (5)	0.90139 (16)	0.89023 (10)	0.0325 (3)	
C4	0.05934 (6)	1.02649 (18)	0.90933 (11)	0.0281 (3)	
C3	0.00567 (6)	1.02975 (19)	0.87524 (11)	0.0319 (4)	
H3	−0.0092	0.9435	0.8402	0.038*	
C7	0.15214 (6)	0.93324 (19)	0.89292 (13)	0.0353 (4)	
H7A	0.1605	1.0164	0.9406	0.042*	
H7B	0.1625	0.9737	0.8306	0.042*	
C6	0.18440 (6)	0.78141 (18)	0.91761 (12)	0.0338 (4)	
H6A	0.2226	0.8049	0.9141	0.041*	
H6B	0.1774	0.7484	0.9831	0.041*	
C10	0.16963 (6)	0.64399 (17)	0.84886 (11)	0.0279 (3)	
H10	0.1788	0.6761	0.7834	0.033*	
C9	0.10936 (6)	0.6144 (2)	0.85116 (13)	0.0363 (4)	
H9A	0.1003	0.5765	0.9147	0.044*	
H9B	0.0993	0.5304	0.8050	0.044*	
C2	−0.02438 (6)	1.1647 (2)	0.89536 (11)	0.0329 (4)	
N3	0.06371 (7)	1.37851 (19)	1.05091 (12)	0.0416 (4)	
C8	0.07765 (7)	0.7682 (2)	0.82731 (13)	0.0403 (4)	
H8A	0.0832	0.7984	0.7608	0.048*	
H8B	0.0395	0.7476	0.8341	0.048*	
C1	0.04550 (6)	1.26709 (18)	0.98595 (11)	0.0307 (3)	
C12	0.26044 (7)	0.5144 (2)	0.85548 (14)	0.0407 (4)	
H12A	0.2740	0.6127	0.8844	0.061*	
H12B	0.2641	0.5195	0.7869	0.061*	
H12C	0.2806	0.4236	0.8810	0.061*	
C11	0.18204 (7)	0.34164 (19)	0.83246 (13)	0.0397 (4)	
H11A	0.1446	0.3278	0.8462	0.059*	
H11B	0.2024	0.2515	0.8582	0.059*	
H11C	0.1860	0.3468	0.7639	0.059*	
C5	−0.08123 (8)	1.1816 (3)	0.85504 (15)	0.0527 (5)	
H5A	−0.0969	1.2794	0.8792	0.079*	
H5B	−0.1022	1.0901	0.8743	0.079*	
H5C	−0.0808	1.1862	0.7859	0.079*	

### Atomic displacement parameters (Å^2^)

**Table:**

	*U*^11^	*U*^22^	*U*^33^	*U*^12^	*U*^13^	*U*^23^
Cl1	0.0423 (3)	0.0540 (3)	0.0307 (2)	−0.00239 (18)	−0.00195 (18)	0.00571 (17)
N1	0.0286 (7)	0.0259 (7)	0.0397 (7)	0.0011 (5)	0.0026 (5)	−0.0023 (5)
N5	0.0331 (7)	0.0263 (7)	0.0263 (7)	0.0036 (5)	0.0014 (5)	0.0005 (5)
N2	0.0335 (7)	0.0315 (7)	0.0399 (7)	0.0060 (6)	0.0011 (6)	−0.0022 (6)
N4	0.0275 (7)	0.0266 (7)	0.0434 (8)	0.0023 (5)	−0.0001 (6)	−0.0071 (6)
C4	0.0307 (8)	0.0251 (7)	0.0286 (7)	0.0013 (6)	0.0045 (6)	0.0022 (6)
C3	0.0334 (8)	0.0325 (8)	0.0296 (8)	0.0027 (6)	−0.0014 (6)	−0.0032 (6)
C7	0.0287 (8)	0.0255 (8)	0.0518 (10)	−0.0005 (6)	0.0050 (7)	−0.0019 (7)
C6	0.0276 (8)	0.0267 (8)	0.0470 (9)	0.0009 (6)	−0.0017 (7)	−0.0061 (7)
C10	0.0337 (8)	0.0234 (7)	0.0266 (7)	0.0037 (6)	0.0015 (6)	0.0019 (6)
C9	0.0345 (9)	0.0266 (8)	0.0474 (10)	0.0001 (7)	−0.0048 (7)	−0.0086 (7)
C2	0.0319 (8)	0.0370 (9)	0.0299 (8)	0.0052 (7)	0.0010 (6)	0.0007 (7)
N3	0.0348 (8)	0.0326 (8)	0.0571 (9)	0.0052 (7)	−0.0033 (7)	−0.0124 (7)
C8	0.0356 (9)	0.0351 (9)	0.0494 (10)	0.0066 (7)	−0.0094 (7)	−0.0121 (8)
C1	0.0314 (8)	0.0261 (8)	0.0348 (8)	0.0002 (6)	0.0055 (6)	0.0006 (6)
C12	0.0325 (9)	0.0385 (9)	0.0510 (10)	0.0050 (7)	0.0022 (7)	−0.0055 (8)
C11	0.0431 (9)	0.0241 (8)	0.0516 (10)	0.0029 (7)	0.0004 (8)	−0.0036 (7)
C5	0.0419 (10)	0.0593 (12)	0.0559 (12)	0.0167 (9)	−0.0140 (9)	−0.0128 (10)

### Geometric parameters (Å, º)

**Table:**

N1—C1	1.340 (2)	C10—C9	1.515 (2)
N1—C4	1.342 (2)	C10—H10	0.9800
N5—C11	1.488 (2)	C9—C8	1.522 (2)
N5—C12	1.490 (2)	C9—H9A	0.9700
N5—C10	1.5196 (19)	C9—H9B	0.9700
N5—H5N	0.878 (19)	C2—C5	1.505 (2)
N2—C2	1.341 (2)	N3—C1	1.353 (2)
N2—C1	1.347 (2)	N3—H3NA	0.85 (2)
N4—C4	1.378 (2)	N3—H3NB	0.84 (2)
N4—C8	1.453 (2)	C8—H8A	0.9700
N4—C7	1.458 (2)	C8—H8B	0.9700
C4—C3	1.397 (2)	C12—H12A	0.9600
C3—C2	1.373 (2)	C12—H12B	0.9600
C3—H3	0.9300	C12—H12C	0.9600
C7—C6	1.518 (2)	C11—H11A	0.9600
C7—H7A	0.9700	C11—H11B	0.9600
C7—H7B	0.9700	C11—H11C	0.9600
C6—C10	1.518 (2)	C5—H5A	0.9600
C6—H6A	0.9700	C5—H5B	0.9600
C6—H6B	0.9700	C5—H5C	0.9600
			
C1—N1—C4	116.57 (13)	C8—C9—H9A	109.4
C11—N5—C12	108.81 (12)	C10—C9—H9B	109.4
C11—N5—C10	113.97 (12)	C8—C9—H9B	109.4
C12—N5—C10	111.73 (12)	H9A—C9—H9B	108.0
C11—N5—H5N	106.4 (12)	N2—C2—C3	122.82 (14)
C12—N5—H5N	107.3 (12)	N2—C2—C5	116.69 (14)
C10—N5—H5N	108.3 (12)	C3—C2—C5	120.49 (15)
C2—N2—C1	115.07 (13)	C1—N3—H3NA	119.1 (14)
C4—N4—C8	120.92 (13)	C1—N3—H3NB	118.5 (13)
C4—N4—C7	118.99 (13)	H3NA—N3—H3NB	116.1 (19)
C8—N4—C7	114.18 (13)	N4—C8—C9	111.39 (13)
N1—C4—N4	116.06 (13)	N4—C8—H8A	109.3
N1—C4—C3	120.80 (14)	C9—C8—H8A	109.3
N4—C4—C3	123.14 (14)	N4—C8—H8B	109.3
C2—C3—C4	117.64 (15)	C9—C8—H8B	109.3
C2—C3—H3	121.2	H8A—C8—H8B	108.0
C4—C3—H3	121.2	N1—C1—N2	126.69 (14)
N4—C7—C6	111.57 (13)	N1—C1—N3	116.72 (14)
N4—C7—H7A	109.3	N2—C1—N3	116.58 (14)
C6—C7—H7A	109.3	N5—C12—H12A	109.5
N4—C7—H7B	109.3	N5—C12—H12B	109.5
C6—C7—H7B	109.3	H12A—C12—H12B	109.5
H7A—C7—H7B	108.0	N5—C12—H12C	109.5
C10—C6—C7	111.07 (13)	H12A—C12—H12C	109.5
C10—C6—H6A	109.4	H12B—C12—H12C	109.5
C7—C6—H6A	109.4	N5—C11—H11A	109.5
C10—C6—H6B	109.4	N5—C11—H11B	109.5
C7—C6—H6B	109.4	H11A—C11—H11B	109.5
H6A—C6—H6B	108.0	N5—C11—H11C	109.5
C9—C10—C6	108.89 (12)	H11A—C11—H11C	109.5
C9—C10—N5	112.52 (12)	H11B—C11—H11C	109.5
C6—C10—N5	108.95 (12)	C2—C5—H5A	109.5
C9—C10—H10	108.8	C2—C5—H5B	109.5
C6—C10—H10	108.8	H5A—C5—H5B	109.5
N5—C10—H10	108.8	C2—C5—H5C	109.5
C10—C9—C8	111.32 (14)	H5A—C5—H5C	109.5
C10—C9—H9A	109.4	H5B—C5—H5C	109.5

### Hydrogen-bond geometry (Å, º)

**Table:**

*D*—H···*A*	*D*—H	H···*A*	*D*···*A*	*D*—H···*A*
N3—H3*NB*···Cl1	0.84 (2)	2.60 (2)	3.4284 (17)	167.2 (17)
N5—H5*N*···Cl1^i^	0.878 (19)	2.20 (2)	3.0785 (14)	177.1 (17)
N3—H3*NA*···N2	0.86 (2)	2.26 (2)	3.114 (2)	175.5 (18)

Symmetry code: (i) *x*, *y*−1, *z*.
